# Application of new eco-friendly LCMs for combating the lost circulation in heavy-weight and oil-based mud

**DOI:** 10.1039/c7ra13668d

**Published:** 2018-03-06

**Authors:** Alireza Nasiri, Mohammadjavad Ameri Shahrabi, Mostafa Keshavarz Moraveji

**Affiliations:** Department of Petroleum Engineering, Amirkabir University of Technology (Tehran Polytechnic) 424 Hafez Avenue Tehran 15875-4413 Iran; Department of Chemical Engineering, Amirkabir University of Technology (Tehran Polytechnic) 424 Hafez Avenue Tehran 15875-4413 Iran moraveji@aut.ac.ir

## Abstract

This study investigates the effectiveness of different lost circulation materials (LCMs) in controlling the lost circulation of heavy-weight and oil-based mud. The bridging material tester (BMT) and three-dimensional fractures were used to evaluate the fracture sealing performance of different additives. Two new eco-friendly LCMs, namely, RIPI-LQ and X1-Seal were used in the present study. RIPI-LQ was made from a special type of grass and X1-Seal additive was made from a flowering plant. Due to eco-friendly characteristics of these two additives, the risk of environmental effect was reduced. The experimental results clearly indicated that the performance of these new additives was superior to widely used LCMs such as Quick Seal, mica, oyster shell and walnut shell. Finally, the results of this study were validated using a field test. The field test results demonstrated that these new eco-friendly LCMs were able to control different types of lost circulation.

## Abstract 2

This study investigates the effectiveness of different lost circulation materials (LCMs) in controlling the lost circulation of heavy-weight and oil-based mud.

## Graphical abstract

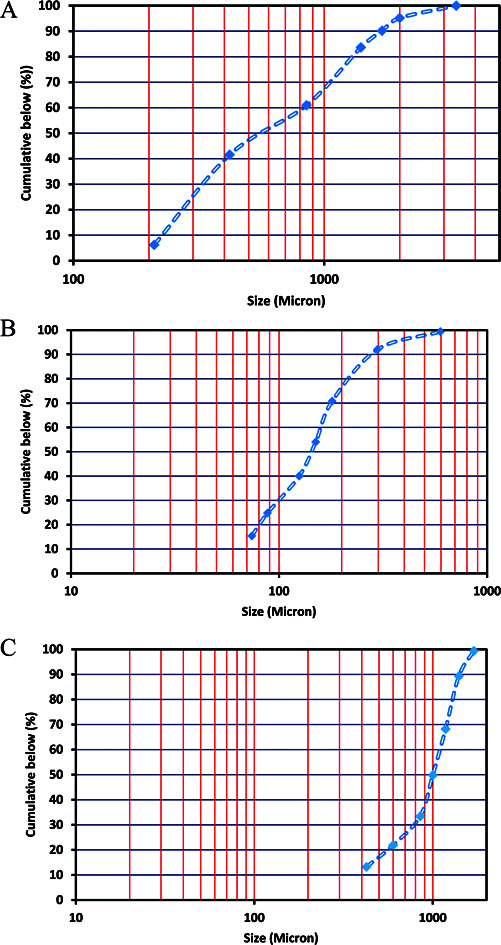

## 1. Introduction

The lost circulation of drilling fluids is one of the most notorious problems of the drilling industry, which frequently occurs in highly permeable formations, cave-like beds and formations with inherent or induced fractures.^1,2^ Generally, the lost circulation of drilling fluids is categorized into three groups: complete, partial and seeping loss.^3^ If the rate of lost circulation is higher than 500 barrel per hour (bph), it is considered as a complete loss. Long horizontal and vertical fractures, vertical fractures with large openings, big voids and other highly permeable zones are considered primarily responsible for this type of lost circulation.^4^ Lost circulation with rates between 10 to 500 bph is called partial loss, which primarily occurs in small natural fractures, gravels and vertical fractures with small openings.^4^ Finally, the seeping loss category is used to define light losses with the rate of up to around 10 bph. This type of lost circulation can be easily controlled by the reduction or stoppage of mud pumping and allowing the fractures to be filled by the solid portion of drilling mud.^3,4^

Lost circulation causes several problems, such as the loss of several barrels of drilling fluids, increase in the non-productive rig time, loss in the wellbore, and in some cases even blowouts.^5,6^ It is also blamed for the excessive caving of formations, which in turn results in cement job problems and reduces the effective permeability of the near wellbore region. The excessive cost of these types of problems is many million dollars annually.^4,7^

Different lost circulation control techniques, such as adding LCMs to the drilling mud, wellbore strengthening, and pneumatic drilling can be used to combat or prevent lost circulation of drilling fluids.^8–10^ Wellbore strengthening method is defined as the process of isolating the fractures from wellbore fluids and controlling the fracture propagation.^10^ Pneumatic drilling fluids comprise of three general groups: air/gas, aerated fluid, and foams. When using these drilling fluids, specific equipment such as compressors, tanks, lines and valves are needed to guarantee the safety of the drilling operation.^11^ Furthermore, advanced drilling technology such as expandable tubular technology and casing while drilling can be considered as a new option to mitigate the risk of lost circulation in naturally fractured or highly permeable reservoirs. However, these methods are not available in all countries and require more expensive equipment.^12,13^

Among all the above mentioned techniques, the most outstanding method to treat and prevent the occurrence of the lost circulation phenomenon is the use of LCMs. Commonly used LCMs can be categorized into four general groups: granular, fibrous, flaky, or a blend of all three.^4,7^ Howard *et al.* used granular LCMs to seal small fractures. Their experimental results indicated that the concentration of an LCM is a controlling factor affecting the ability of the material to plug fractures.^14^ Loeppke *et al.* used granular LCMs such as Gilsonite, mixed nut shells and perlite to seal fractures. They concluded that the particle size distribution of LCMs is an important factor in the fracture plugging process.^15^

Routinely, fibrous materials are used to control lost circulation in highly permeable porous formations. McKinley and Applegath used a fibrous LCM to prevent lost circulation. In that study, the LCM pills consisted of fibrous and polymeric absorbers, which swelled due to the liquid around them.^16^ Another type of LCM is the flaky type. These materials are used to block fractures and large voids.^4,7^

There are also some studies that used a blend of fibrous, flaky and granular LCMs to combat heavy losses. Pilehvari and Nyshadham studied various blends of granular, fibrous, and flaky LCMs and determined the effect of size distribution on their performance.^17^ Whitfill and Hemphill used a combination of resilient graphite carbon and sized calcium carbonate to mitigate the lost circulation of oil-based mud.^18^ Goud and Joseph used a blend of crystalline graphite and calcium carbonate to plug fractures. They explained that the crystalline graphite enters small fractures and forms an initial seal, while the calcium carbonate forms an external bridge at the fracture mouth.^19^

The particle size distribution is an important factor affecting the ability of LCMs to seal fractures, loose sands and gravels. There are several publications that focused on the particle size distribution of LCMs. Cargnel and Luzardo studied the particle size distribution of calcium carbonate. They concluded that as long as the particle size distribution of calcium carbonate is within the range of 1/7 and 1/3 of the average pore throat size, it can effectively seal porous formations.^20^ Dick *et al.* discussed the ideal packing theory (IPT). In this method, based on the formation characteristics, a linear graph is used to determine the optimum particle size distribution of LCMs.^21^ Vickers *et al.* presented a new criterion to optimize the particle size distribution of LCMs based on the reservoir pore throat distribution.^22^ Alsaba *et al.* presented a method to select the optimum particle size distribution for effective fracture sealing. Their experimental results indicated that if the D50 and D90 dispersion parameters of LCMs are equal to or greater than 3/10 and 6/5 fracture width, respectively, the amount of lost circulation will be reduced.^23^ Based on the above mentioned studies, it can be concluded that optimizing the particle size distribution of LCMs is an important aspect of combating lost circulation.

Over the last few years, several eco-friendly LCMs have been developed to reduce the environmental impacts of loss controllers.^24,25^ Burts used different rice fractions, such as rice hulls, rice tips, rice straw and rice bran to mitigate the lost circulation.^26^ Cremeans *et al.* used cotton seed hulls as an environmentally friendly LCM. This additive also improves the bit lubrication.^27^ MacQuoid and Skodack used coconut coir to prevent the loss of drilling fluid.^28^ Weaver introduced a wood-based additive to control the lost circulation. This eco-friendly additive can be screened to different sizes and used in various drilling operations.^29^

In the present study, a BMT (bridging material test) apparatus and 5 cm-depth slots were used to determine the effectiveness of various LCMs in heavy-weight and oil-based mud. The properties of the drilling fluids and LCMs used were specified based on field information. Two new eco-friendly additives were also used to control the lost circulation of various sized fractures. The results indicated that these new eco-friendly additives have better performance than their toxic counterparts. Lastly, these new additives were used to control the lost circulation in one of the Iranian southern oil fields. The results of this field test indicated that the LCM pills used are well capable of combating medium and heavy losses.

## 2. Experimental

### 2.1 Materials

According to the data related to different wells drilled in Iran, two types of drilling fluids were handpicked for the practical experiments of this study. The first fluid is the heavy-weight mud, which is mostly used to drill the Gachsaran formation. This formation is plastic and charged with high-pressure formation water. There are some instances where mud weights of 165 (pcf) have been used to drill through the upper Gachsaran layers. Horizontal and vertical fractures, large voids and cave-like beds exist in this formation. Thus, partial and even complete losses usually occur in the Gachsaran formation.

The other important drilling fluid, which was used in the practical experiments, was oil-based mud. This type of drilling fluid is routinely used to drill production zones, water soluble zones and troublesome shale formations.^30^ In the south of Iran, oil-based mud is mostly used to drill production formations (Fahlian, Asmari and Sarvak) and shale formations (Pabdeh, Gurpi and Kajdomi). Several fractures exist in these formations, which may cause partial or heavy losses. This clearly demonstrates the importance of investigating the lost circulation of oil-based mud. The properties of the used drilling fluids are presented in Table 1.

**Table 1:** Properties of heavy-weight and oil-based drilling fluids

	Oil-based mud (oil/water ratio: 70/30)	Heavy-weight mud
Apparent viscosity (cP)	16.5	75
Plastic viscosity (cP)	13	65
Yield point (lb/100 ft^2^)	7	20
Mud weight (pcf)	65	135

Two new eco-friendly LCMs (RIPI-LQ and X1-Seal) were used to control different types of lost circulation. RIPI-LQ was made from one of the grass species that grows in the Zagros Mountains. The amount of heat used to remove moisture from this species has a direct impact on its ability to form a stable bridge. It should be emphasized that based on the particle size distribution, RIPI-LQ was categorized into two types: RIPI-LQC (RIPI-LQ coarse) and RIPI-LQF (RIPI-LQ fine). X1-Seal was made from an economically important type of flowering plant, which belongs to the Brassicaceae family. Several parameters such as the particle size distribution, the amount of moisture and the method used to grind this plant have a great impact on its fracture sealing performance.

**Fig. 1:**
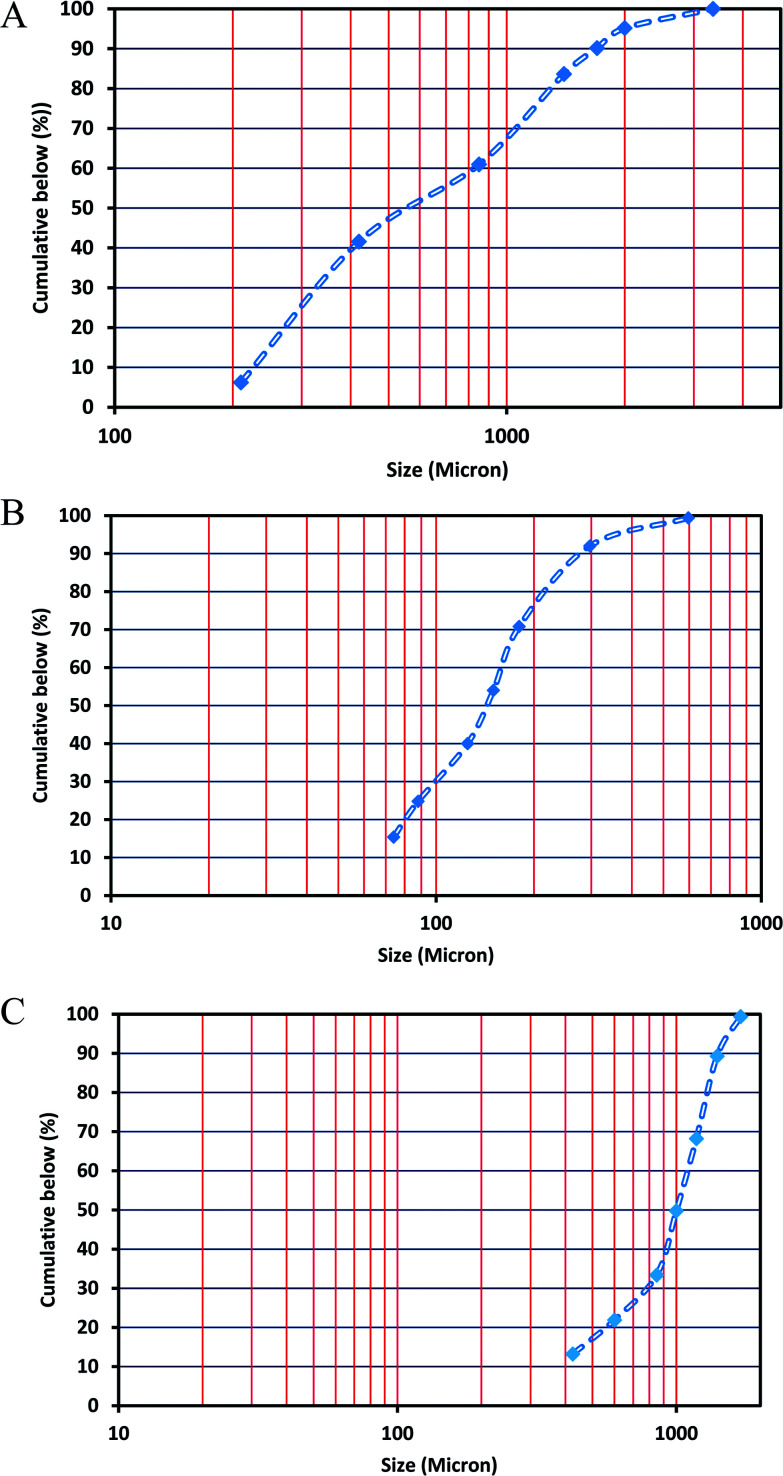
Particle size distribution of RIPI-LQF (A), RIPI-LQC (B) and X1-Seal (C).

As mentioned before, the performance of these new eco-friendly additives was compared with several well-known commercial LCMs, such as walnut shell, Quick Seal, mica and oyster shell. The physical properties and particle size distribution of each of these LCMs are presented in Table 2. To determine the solubility of the above mentioned LCMs in acid, 10 g of each additive were precisely weighed and added to 100 mL of 28% hydrochloric acid. Standard ceramic sieves were then used to determine the amount of additives that could no longer dissolve in the acid. In addition, ASTM E11 sieves were used to determine the particle size distribution of LCMs. For this purpose, a specific amount of each LCM was poured onto the sieves and sieved for 30 min using a shaker. Then, the percentage of materials passing through each sieve was calculated.^7^ The results of this investigation for RIPI-LQC, RIPI-LQF and X1-Seal are shown in Fig. 1.

**Table 2:** Physical properties and particle size distribution of used LCMs

LCM	Minimum size (micron)	Maximum size (micron)	Specific gravity	Solubility in acid (%)	Other properties
Oyster shell (coarse)	2360	9500	2.83	97	Because of the high solubility in acid, after acidizing, this additive has almost no effect on the permeability of the reservoirs
Walnut shell (coarse)	1700	8000	1.4	0.11	This LCM is a hard additive made from crushed walnut shell
Mica (coarse)	3350	12 500	2.8	3	Layered gray material-generally inert material with no reactions to hydrocarbons, acids, brines
Quick Seal (coarse)	180	2000	2.22	12	Gray powdery material – with wide particle size distribution – this material is a mixture of layered loss inhibitors (mica) and hull of herbs
RIPI-LQF (fine)	74	595	1.68	1	A type of gray cellulosic material – biodegradable – this material makes the movement of drill string and logging instruments easier and smoother
RIPI-LQC (coarse)	210	3360			
X1-Seal	450	1700	1.36	25	Stable in high temperature – biodegradable – this material also reduces the amount of drilling mud filtration and enhance the filter cake

### 2.2 Apparatus setup

**Fig. 2:**
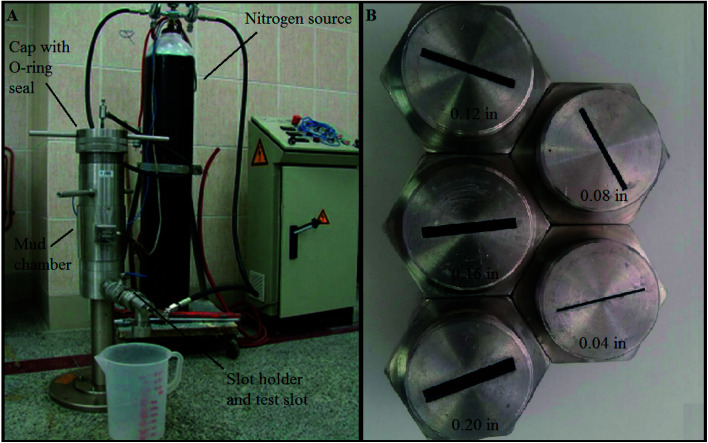
Schematic of BMT apparatus (A) and BMT slots (B).^7^

The schematic of the BMT apparatus is shown in Fig. 2A. In this apparatus, three-dimensional slots, each of which was 1.96 inches in depth, 1.38 inches in length, and 0.04, 0.08, 0.12, 0.16, and 0.2 inches in width were used (Fig. 2B).^7^ Based on the slots' dimensions, the flow rates of heavy-weight and oil-based mud at 1000 psi were calculated for different slots of the BMT apparatus, as presented in Table 3. The permeability of each slot was also determined and presented in this table. The flow rate of oil-based mud at 1000 psi was higher than that at 500 bph for fractures that were 0.12, 0.16 and 0.2 inches in width. In addition, the flow rates of heavy-weight mud in 0.16 and 0.2 inch-wide fractures were also greater than those at 500 bph. As a result, the following experiments performed on these sizes of fractures can be used to model complete losses. It should be emphasized that previously, many research groups have performed similar experiments using fractures without depth, while the fractures used in the present study have depth. Using this type of fracture makes the conditions much more similar to those of real wells. This is primarily because if an LCM can block a fracture from inside, it could be applicable in these fractures.^7^

**Table 3:** Calculated permeabilities and flow rates of heavy-weight and oil-based drilling fluids at 1000 psi for different slots of BMT apparatus

Fracture opening (in)	Fracture permeability (darcy)	Oil-based mud	Heavy-weight mud
0.04	2522.9	10.61	2.9
0.08	20 183.1	169.69	46.44
0.12	68 118.2	859.05	235.11
0.16	161 465.5	2715.04	743.06
0.2	315 362.3	6628.50	1814.12

### 2.3 Measurement method

In this research, the API 13I (American Petroleum Institute) standard method was used with the BMT apparatus to evaluate the amount of lost circulation.^31^ For analysis, slots were placed before the output valve. Drilling mud with specific amounts of LCM was then poured into the BMT cell (with the output valve open) and the output mud volume was measured accurately. In the next step, the piston was placed on the mud and the mud pressure was increased by 50 psi at every 10 s intervals. It is necessary to note that the pressure was increased until either 1000 psi was attained or until the stoppage of mud flow. In the cases where LCMs had succeeded in blocking the output passage of the flow, the pressure was kept constant for 10 minutes and then, the final output volume was recorded. Finally, the experiments were repeated after changing the slots (increasing their size) until the permanent blockage at 1000 psi was achieved and the results were used to investigate the performance of various LCMs.

Before presenting the simulation methodology, it is necessary to note that for these tests, there was 3500 milliliters of fluid inside the BMT apparatus cell. Thus, the loss of 3500 milliliters of fluid, indicates that the additive was unable to control the lost circulation. In addition, based on the API guidelines, the performance of an LCM is considered to be very good if it can limit the mud loss to 1000 milliliters or less.

## 3. Results and discussion

### 3.1 The blockage ability of different LCMs in a 0.04 inch-width slot

**Fig. 3:**
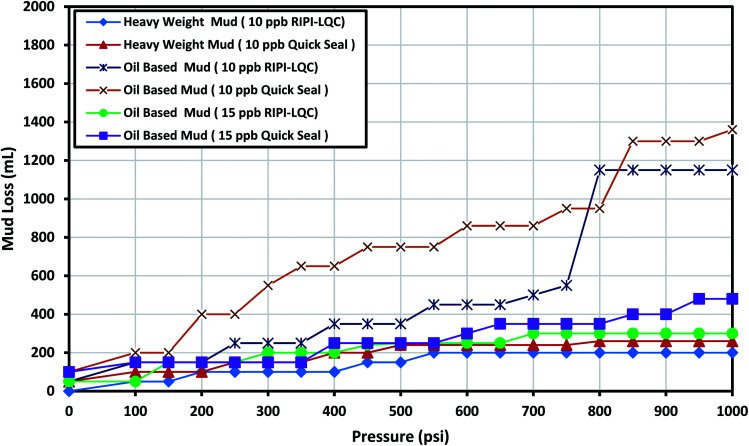
The investigation of performance of RIPI-LQC and Quick Seal to control the lost circulation in heavy-weight and oil-based drilling fluids (0.04 in slot).

This fracture could be used as a decent basis to investigate light losses in oil-based and heavy-weight mud. Fig. 3 shows the results obtained for RIPI-LQC and Quick Seal additives. The results indicated that 15 ppb of RIPI-LQC and Quick Seal were well capable of controlling the lost circulation of the oil-based mud. In addition, 10 ppb of RIPI-LQC and Quick Seal were used to control the lost circulation of the heavy-weight mud. The results also indicated that the performance of RIPI-LQC was higher than that of Quick Seal additive since the amount of fluid loss for RIPI-LQC was less than that for Quick Seal. It should also be emphasized that controlling the lost circulation of the heavy-weight mud was easier than the oil-based mud. This was primarily because lower amounts of RIPI-LQC and Quick Seal were used to combat the lost circulation of heavy-weight mud.

**Fig. 4:**
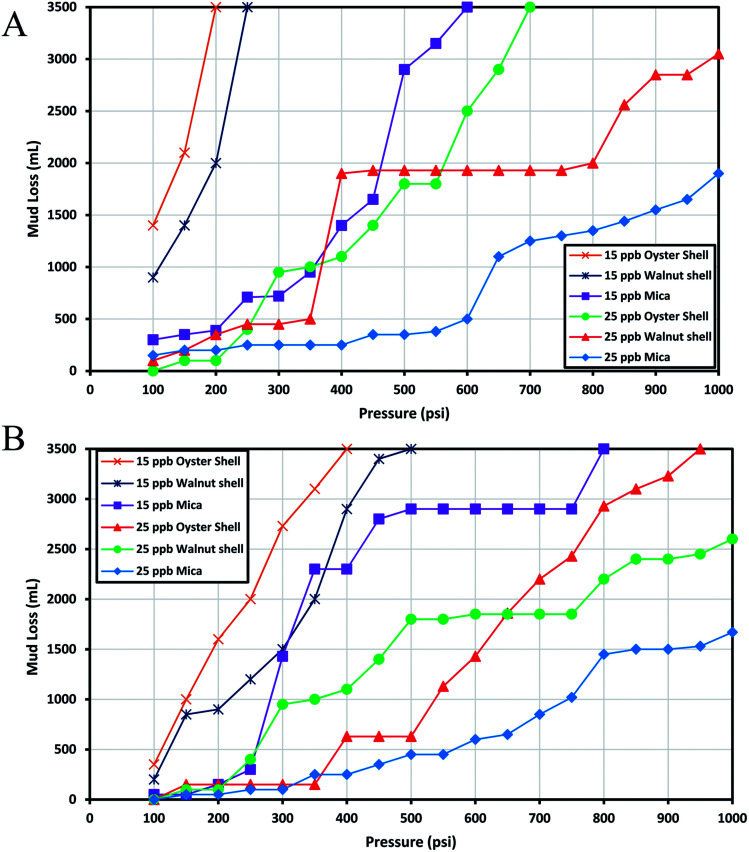
The investigation of performance of oyster shell, walnut shell and mica to control the lost circulation in oil-based (A) and heavy-weight (B) drilling fluids (0.04 in slot).

The capability of mica coarse, oyster shell coarse and walnut shell coarse to block a 0.04 inch-fracture is shown in Fig. 4. As shown in the figure, even high amounts of these LCMs could not effectively seal this fracture. It should be emphasized that mica coarse, oyster shell coarse and walnut shell coarse are routinely used to combat different types of lost circulation in the Bibi Hakimeh, Aghajari and Rag Sefid oil fields. However, the results presented in Fig. 4 clearly indicate that these three additives are not at all appropriate in controlling the lost circulation of fractured reservoirs. This is primarily because of the inappropriate particle size distribution of these additives. The low efficiencies of these LCMs were observed in other sizes of slots too. Therefore, the results of using these three LCMs in other slots are not presented in this study since they cannot be used to control the lost circulation of heavy-weight and oil-based mud.

**Fig. 5:**
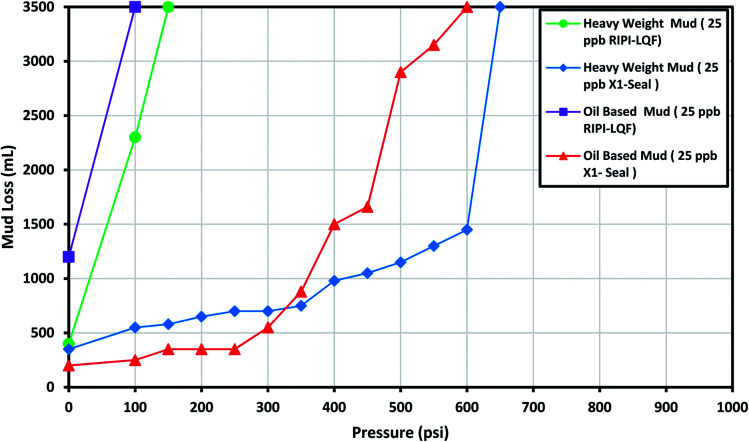
The investigation of performance of RIPI-LQF and X1-Seal to control the lost circulation in heavy-weight and oil-based drilling fluids (0.04 in slot).

The experimental results for X1-Seal and RIPI-LQF are shown in Fig. 5. The results demonstrated that the X1-Seal and RIPI-LQF additives were not able to control the lost circulation of oil-based and heavy-weight mud. The fine particle size of these LCMs is the main cause of this problem. However, in more open fractures, these additives could be used to improve the efficiency of LCM pills by filling the voids between larger LCMs.

### 3.2 The blockage ability of different LCMs in a 0.08-inch width slot

**Fig. 6:**
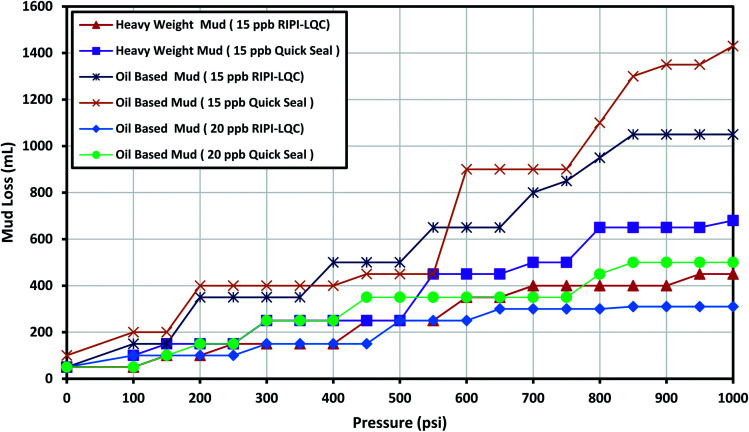
The investigation of performance of RIPI-LQC and Quick Seal to control the lost circulation in heavy-weight and oil-based drilling fluids (0.08 in slot).

This fracture could be used to model light to medium losses. The experimental outcomes for RIPI-LQC and Quick Seal are shown in Fig. 6. It could be seen that 15 ppb of RIPI-LQC and Quick Seal could effectively control the lost circulation of heavy-weight mud. However, to control the lost circulation of the oil-based mud, 20 ppb of these two additives were used. Again, controlling the lost circulation of heavy-weight mud was easier than that of oil-based mud since lower amounts of LCMs were used for this purpose. The data shown in Fig. 6 also indicates that the amount of fluid loss for RIPI-LQC was less than that for Quick Seal in both heavy-weight and oil-based mud. Thus, it can be concluded that the fracture sealing performance of RIPI-LQF is higher than that of Quick Seal.

### 3.3 The blockage ability of different LCMs in a 0.12-inch width slot

**Fig. 7:**
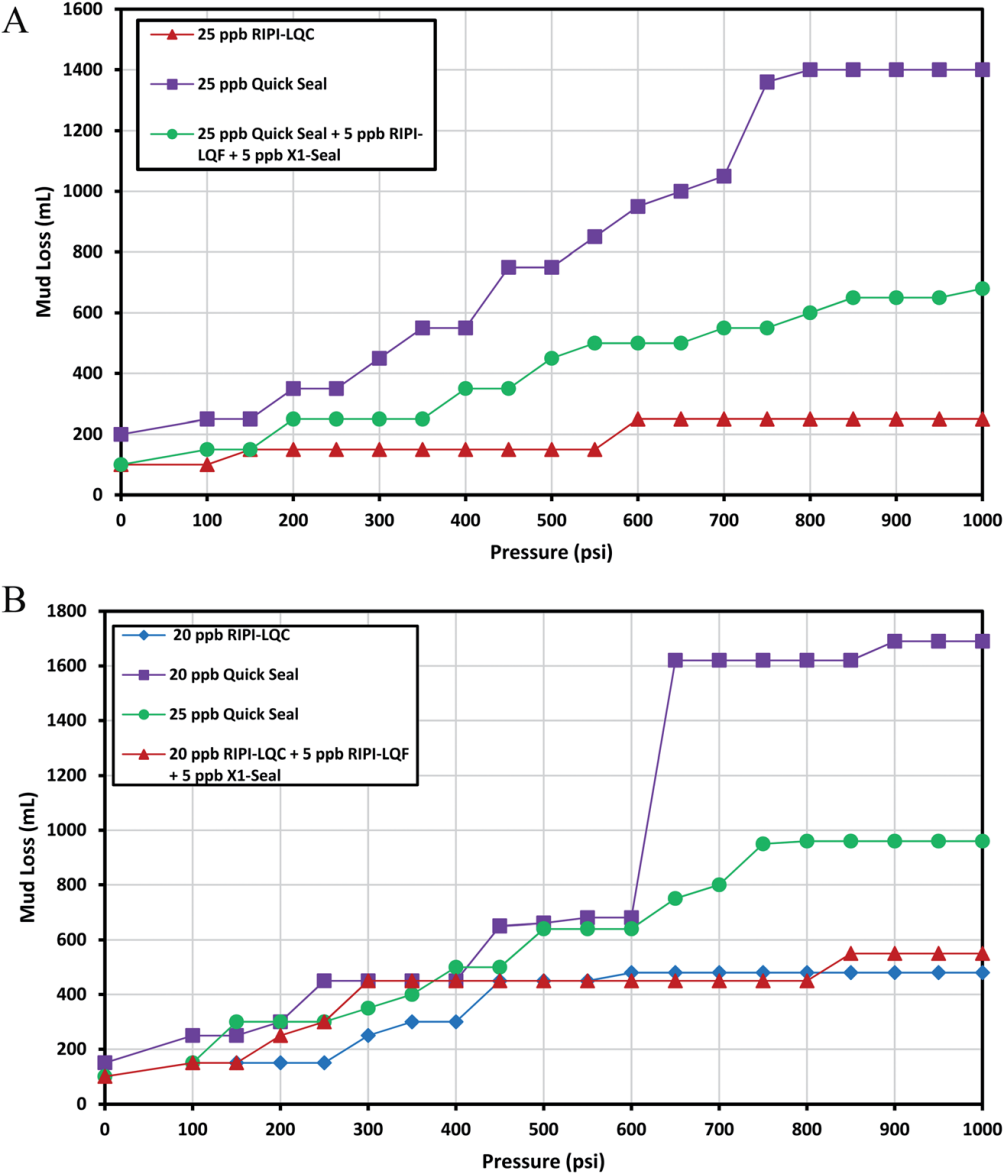
The investigation of performance of RIPI-LQC and Quick Seal to control the lost circulation in oil-based (A) and heavy-weight (B) drilling fluids (0.12 inch slot).

This fracture could be used as a decent basis to investigate medium and heavy losses. The experimental results for controlling the lost circulation of the oil-based mud in 0.12 inch-width fractures are shown in Fig. 7A. The results indicated that 25 ppb of RIPI-LQC exhibited an acceptable performance. However, the same amount of Quick Seal was not able to effectively seal this fracture. As a result, a blend of 25 ppb of Quick Seal, 5 ppb of X1-Seal and 5 ppb of RIPI-LQF was used to control the lost circulation of the oil-based mud in a 0.12 inch-wide fracture. This again demonstrated that small LCMs could be used to improve the efficiency of LCM pills by filling the voids between larger LCMs.

As shown in Fig. 7B, 20 ppb of RIPI-LQC has shown a perfect performance in controlling the lost circulation of heavy-weight mud. When 20 ppb of Quick Seal was used, a jump in the amount of fluid lost was observed at 600 psi. This is primarily because the LCM-bridge was broken at this pressure. Also, the LCM-bridge was formed again at 650 psi and the amount of fluid loss for this amount of Quick Seal was larger than 1000 mL. Therefore, 20 ppb of Quick Seal could not effectively seal this fracture. Consequently, 25 ppb of Quick Seal was used to control the lost circulation of the heavy mud. In another experiment, a blend of 20 ppb of Quick Seal, 5 ppb of X1-Seal and 5 ppb of RIPI-LQF was used to control the lost circulation of heavy-weight mud. The results indicated that the performance of the above mentioned mixture of LCMs was higher than that of 25 ppb of Quick Seal. This is primarily because the combination of Quick Seal, X1-Seal and RIPI-LQF has a more appropriate particle size distribution, such that larger particles formed bridges, while the smaller particles filled the pores and voids between them to properly control the loss. It should be pointed out that controlling the lost circulation of heavy-weight mud was easier than that of oil-based mud in the 0.12 inch-width fracture. This was primarily because a smaller amount of LCMs was used to control the lost circulation of heavy-weight mud compared to the oil-based mud.

### 3.4 The blockage ability of different LCMs in a 0.16 inch-width slot

**Fig. 8:**
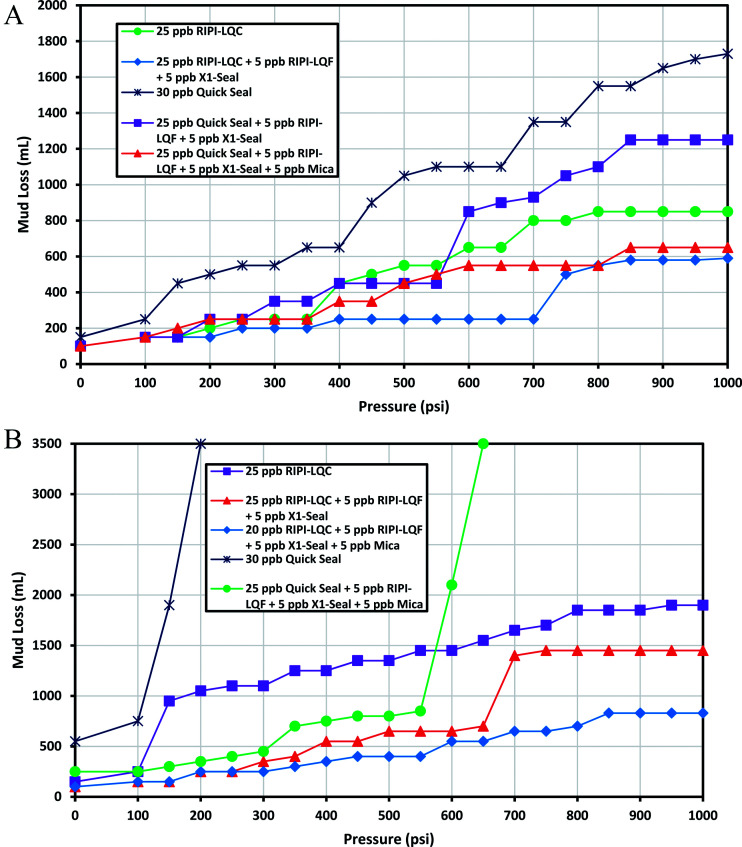
The investigation of performance of RIPI-LQC and Quick Seal to control the lost circulation in oil-based (A) and heavy-weight (B) drilling fluids (0.16 inch slot).

This fracture could be used to model heavy losses. The experimental results for controlling the lost circulation of oil-based mud are shown in Fig. 8A. The results indicated that 25 ppb of RIPI-LQC was able to adequately control the lost circulation of oil-based mud. It should be emphasized that when 5 ppb of RIPI-LQF and 5 ppb of X1-Seal were added to 25 ppb of RIPI-LQC, the lost circulation of oil-based drilling mud was reduced to 590 mL. On the other hand, even 30 ppb of Quick Seal could not seal this fracture. A blend of 25 ppb of Quick Seal, 5 ppb of RIPI-LQF and 5 ppb of X1-Seal was also unable to seal this fracture since the amount of fluid loss for this mixture was larger than 1000 mL. Finally, a combination of 25 ppb of Quick Seal, 5 ppb of RIPI-LQF, 5 ppb of X1-Seal and 5 ppb of mica was used to control the lost circulation of oil-based mud. It is worth mentioning that using the mica additive in these LCM pills increased the maximum particle size of LCMs. Consequently, the particle size distribution of LCMs was more suitable to block such a wide fracture.

Fig. 8B shows the experimental results for controlling the lost circulation of heavy-weight mud in a 0.16 inch-width fracture. As shown in the figure, 25 ppb of RIPI-LQC and a blend of 25 ppb of RIPI-LQC, 5 ppb of RIPI-LQF and 5 ppb of X1-Seal were not able to control the lost circulation of heavy-weight mud. Eventually, a blend of 20 ppb of RIPI-LQC, 5 ppb RIPI-LQF, 5 ppb of X1-Seal and 5 ppb of mica performed adequately to control the loss of heavy-weight mud. The experimental results also indicated that 30 ppb of Quick Seal and a blend of 25 ppb of Quick Seal, 5 ppb RIPI-LQF, 5 ppb of X1-Seal and 5 ppb of mica were not able to control the lost circulation of heavy-weight mud in the 0.16 inch-width fracture. When a blend of 25 ppb of Quick Seal, 5 ppb RIPI-LQF, 5 ppb of X1-Seal and 5 ppb of mica was used, a jump in the amount of fluid-loss was observed at 550 psi. This was primarily because the LCM-bridge was broken at this pressure. However, in this case, the LCM-bridge could not be formed again. Therefore, the blend of 25 ppb of Quick Seal, 5 ppb RIPI-LQF, 5 ppb of X1-Seal and 5 ppb of mica was unable to control the loss and the fluid left the chamber completely.

Comparing the results shown in Fig. 8A and B clearly, we can conclude that controlling the lost circulation of heavy-weight mud was harder than that of oil-based mud in the 0.16 inch width fracture. This is different from the results obtained for 0.04, 0.08 and 0.12 inch-width fractures since in the previous slots, controlling the lost circulation of heavy-weight mud was easier than controlling that of oil-based mud.

### 3.5 The blockage ability of different LCMs in a 0.2-inch width slot

**Fig. 9:**
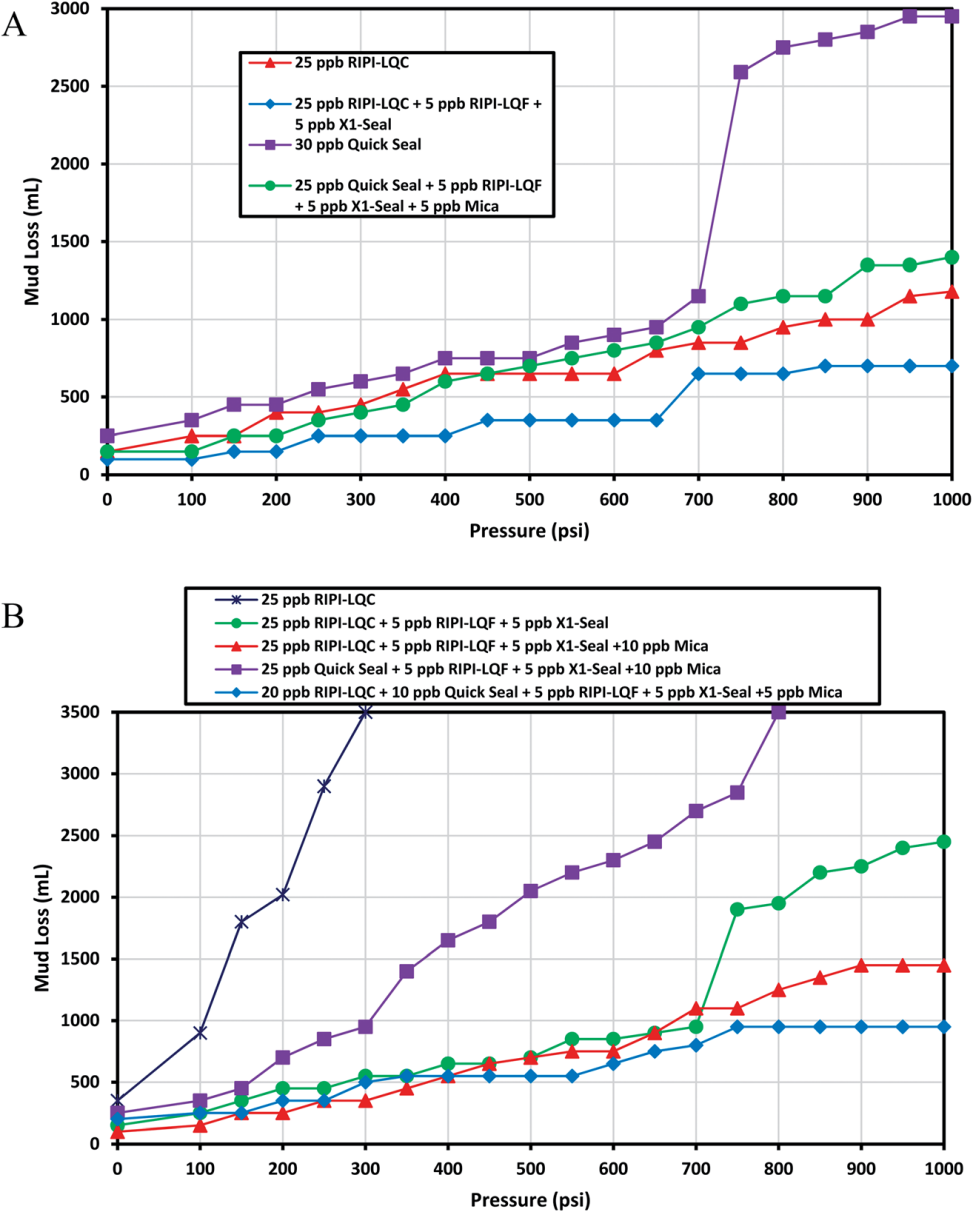
The investigation of performance of RIPI-LQC and Quick Seal to control the lost circulation in oil-based (A) and heavy-weight (B) drilling fluids (0.2 inch slot).

As mentioned before, this fracture at a 1000 psi pressure difference could be a model for heavy losses. Therefore, attention should be paid to the following experiments performed on these sizes of fractures as an approach to control complete losses. As shown in Fig. 9A, 25 ppb of RIPI-LQC could not effectively seal this fracture since the amount of fluid loss for oil-based mud was slightly higher than 1000 mL. However, a blend of 25 ppb of RIPI-LQC, 5 ppb of RIPI-LQF and 5 ppb of X1-Seal could effectively control the lost circulation of oil-based mud. The results also demonstrated that 30 ppb of Quick Seal and a blend of 25 ppb of Quick Seal, 5 ppb of RIPI-LQF, 5 ppb of X1-Seal, 5 ppb of mica were not able to block this fracture.

The experimental results for controlling the lost circulation of heavy-weight mud in a 0.2 inch-width fracture are shown in Fig. 9B. As shown in the figure, controlling the lost circulation of heavy-weight mud in this fracture was really hard. Furthermore, 25 ppb of RIPI-LQC could not form a stable bridge to seal the 0.2 inch-width fracture and hence, the fluid left the chamber completely. A blend of 25 ppb of RIPI-LQC, 5 ppb of RIPI-LQF and 5 ppb of X1-Seal was also ineffective in controlling the lost circulation. In another experiment, a blend of 25 ppb of RIPI-LQC, 5 ppb of RIPI-LQF, 5 ppb of X1-Seal and 10 ppb of mica was used to mitigate the lost circulation. However, this LCM pill was also ineffective due to a high amount of fluid loss. Finally, a combination of 20 ppb RIPI-LQC, 10 ppb Quick Seal, 5 ppb mica, 5 ppb RIP-LQF and 5 ppb X1-Seal was used to control the lost circulation of heavy-weight mud in the 0.2 inch-width fracture. It is worth noting that although the last-mentioned LCM pill could effectively block the 0.2 inch-fracture, it could create excessive circulation pressure in real conditions. In addition, the various additives that are blended this LCM pill could reduce its stability.

The above mentioned results clearly indicated that controlling the lost circulation of heavy-weight mud in a 0.2 inch-width fracture was clearly harder than that of oil-based mud. Comparing the results of this fracture with the previous results, it can be concluded that controlling the lost circulation of heavy-weight mud was harder in 0.2 and 0.16 inch-width fractures, while in 0.04, 0.08 and 0.12 inch-width fractures, controlling the lost circulation of oil-based mud was harder than controlling the lost circulation of heavy-weight mud. In other words, it could be inferred that controlling the heavy losses of heavy-weight drilling mud was more difficult than controlling the loss of oil-based mud. This was in good agreement with the field experience since it is very difficult to mitigate the heavy losses of this type of mud.

## 4. Field test

**Fig. 10:**
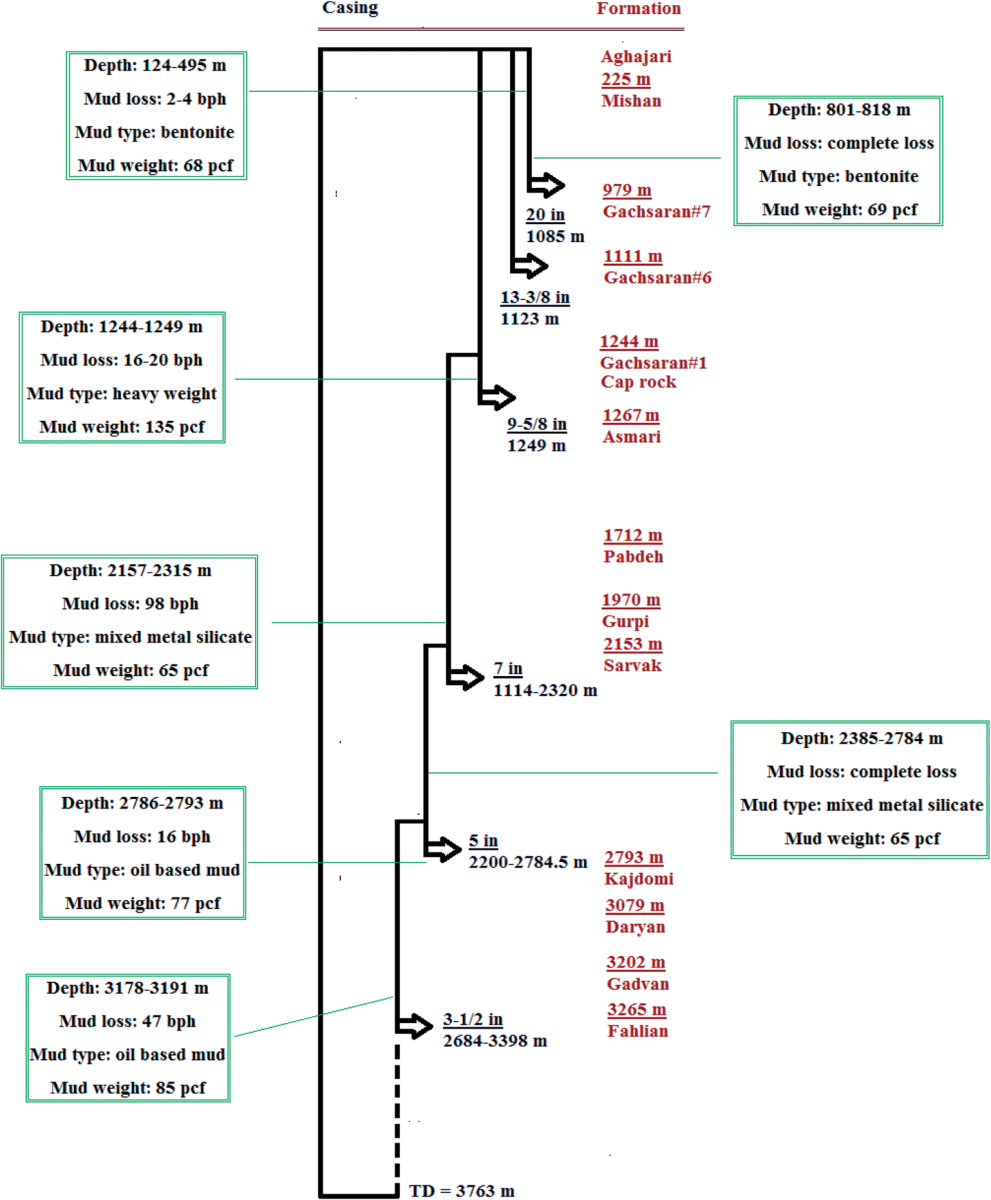
The casing program, the geological profile and the field data of loss controlling process of a production well of Bibi Hakima oil field.

In order to investigate the performance of RIPI-LQC, RIPI-LQF and X1-Seal, different mixtures of these eco-friendly additives were used to control different types of lost circulation in various drilling fluids of a production well. The casing program, the geological profile and the field data of the loss-controlling process of this well are shown in Fig. 10. The loss control techniques used to combat different types of lost circulation in this well are as follows:

• Seeping losses with a rate of 2–4 bph occurred between the depths of 124–495 m. In this case, the mud pumping had been stopped at different depths to allow the fractures to be filled by the solid portion of bentonite mud.

• A complete loss of bentonite mud was observed between the depths of 801–818 m. At first, a cementation process was accomplished to control the lost circulation. After allocating enough time for thickening of the cement, drilling was restarted and the cement was drilled. Again, heavy lost circulation occurred. Following this, a blend of 18 ppb of RIPI-LQC, 7 ppb of RIPI-LQF and 5 ppb of X1-Seal was used to control the lost circulation. After pumping 390 bbl of this LCM pill, the mud loss was reduced significantly to 9 bph.

• A light loss of heavy-weight mud with a rate of 16–20 bph occurred between the depths of 1244–1249 m. Based on the results of this study, 15 ppb of RIPI-LQC was added to heavy-weight mud. After pumping 80 bbl of this LCM pill, no mud loss was observed.

• A medium loss of mixed metal silicate mud with a rate of 98 bph occurred between the depths of 2157–2315 m. A blend of 20 ppb of RIPI-LQC, 5 ppb of RIPI-LQF and 5 ppb of X1-Seal was added to the drilling fluid. In total, 100 barrels of this pill were injected into the well, due to which the mud loss rate reduced to 1 bph. Drilling was continued to the depth of 2320 m with no further mud loss.

• Complete losses of mixed metal silicate mud occurred between the depths of 2385–2784 m. The cementation process was accomplished in different phases to control the lost circulation. Again, heavy lost circulation occurred. In addition, a blend of 25 ppb of RIPI-LQC, 5 ppb of RIPI-LQF, 5 ppb X1-Seal and 5 ppb of mica was still not able to control the lost circulation. Subsequently, the drilling was continued and no fluid was obtained at the surface. Finally, a 5 inch-liner was used to seal this troublesome formation.

• A light loss of oil-based mud with a rate of 16 bph occurred between the depths of 2786–2793 m. Furthermore, 20 ppb of RIPI-LQC was added to oil-based mud and then, 50 bbl of RIPI-LQC pill was injected into the well. Subsequently, 10 ppb of this LCM pill was injected into the well every hour (for 8 hours) and consequently, no further mud loss was observed.

• A medium loss of oil-based mud with a rate of 47 bph occurred between the depths of 3178–3191 m. Again, 20 ppb of RIPI-LQC was used to control the lost circulation. Further, 150 bbl of this LCM pill was injected into the well at different stages, due to which the mud loss reduced significantly to as low as 1 to 2 bph.

## 5. Conclusion

Using the BMT apparatus and three-dimensional slots, the performance of various LCMs in controlling the lost circulation of heavy-weight and oil-based mud was investigated. In addition, two new eco-friendly additives were introduced in this study. The summary of the results acquired by the experimental method in this study and a field test is as follows:

(1) The fracture sealing performance of the new eco-friendly LCMs (RIPI-LQ and X1-Seal) was higher than that of their toxic counterparts.

(2) Although mica coarse, oyster shell coarse and walnut shell coarse additives are routinely used to combat the lost circulation of drilling fluids in Bibi Hakimeh, Aghajari and Rag Sefid oil fields, it was found that they could not even block small fractures.

(3) In 0.04, 0.08 and 0.12 inch-width fractures, controlling the lost circulation of oil-based mud was harder than that of heavy-weight mud.

(4) In 0.2 and 0.16 inch-width fractures, controlling the lost circulation of heavy-weight mud was harder than controlling the lost circulation of oil-based mud.

(5) The field test results demonstrated that a blend of RIPI-LQ and X1-Seal were well capable of controlling the lost circulation of different types of drilling fluids.

## Conflicts of interest

There are no conflicts to declare.

## Supplementary Material
